# Oral administration of glycyrrhizic acid with intramuscular injection of foot-and-mouth disease vaccine enhances the adaptive immune system

**DOI:** 10.3389/fmicb.2025.1502630

**Published:** 2025-02-19

**Authors:** Seokwon Shin, Hyeong Won Kim, Mi-Kyeong Ko, So Hui Park, Jong-Hyeon Park, Su-Mi Kim, Min Ja Lee

**Affiliations:** Center for Foot-and-Mouth Disease Vaccine Research, Animal and Plant Quarantine Agency, Gimcheon, Gyeongsangbuk, Republic of Korea

**Keywords:** oral administration, glycyrrhizic acid, mucosal immunity, systemic immunity, secretory IgA

## Abstract

**Background:**

Commercial foot-and-mouth disease (FMD) vaccines have several drawbacks, including a short duration of the immune response after vaccination and local adverse reactions at the vaccination site. Therefore, we developed a new vaccination strategy that simultaneously improves the health status of the host and stimulates systemic immunity by combining the oral administration of glycyrrhizic acid (GA) and intramuscular injection of the FMD vaccine.

**Methods:**

We evaluated the efficacy of the oral immune enhancer GA in conjunction with an intramuscular injection of the FMD vaccine. After vaccination, the experimental (mice) and target animals (pigs) were orally administered GA daily for 4 weeks and once a week for the next 4 weeks. Subsequently, we evaluated safety using various biochemical serum assays, the efficacy of inducing immune responses using serological assays, and the expression of genes related to systemic immunity induction.

**Results:**

Oral administration of GA in combination with an intramuscular injection of the FMD vaccine enhanced early, mid-term, and long-term immunity in experimental and target animals. We also confirmed that this co-administration increased the expression of secretory IgA (sIgA), an important indicator of mucosal immunity. Additionally, significant gene elevations in systemic immune markers along with T helper (Th) immune responses were observed.

**Conclusion:**

These findings suggest that combining the oral administration of GA with the intramuscular injection of an inactivated FMD vaccine can induce a potent and sustained immune response and stimulate the systemic immune system by promoting sIgA and cytokine gene expression. Our research can be used to enhance the efficacy of existing commercial vaccines as well as control other animal diseases by improving the host’s immune system.

## 1 Introduction

Foot-and-mouth disease (FMD) is a highly contagious veterinary disease listed on the World Organisation for Animal Health (WOAH) list of notifiable animal diseases. Since its identification as a viral disease in 1897, FMD has consistently caused a significant economic burden in endemic regions. In Africa, it leads to annual economic losses exceeding $2 billion, while even in regions or countries free from FMD, it causes losses of over $1.5 billion annually (Knight-Jones and Rushton, 2013). The global market for FMD vaccines, used to control FMD, is predicted to steadily increase, reaching over $2.4 billion by 2031 (Wubshet et al., 2024). It can occur in more than 70 species of hoofed animals, including cattle, pigs, goats, and sheep, and has a substantial impact on the economy and international trade of the countries where it occurs (Jamal and Belsham, 2013). Although FMD vaccination successfully induces antibody production and increases antibody titers, FMD virus (FMDV) outbreaks still occur. This is due to various reasons, such as the short-lived immunity of FMD vaccines and the diverse serotypes of FMDV, which contribute to the continued global incidence of FMD, resulting in substantial economic losses.

To overcome FMD, various studies related to antigen platform development have been conducted, including research on inactivated viruses, virus-like particles, peptides, and DNA and RNA vaccines (Kotla et al., 2016; Xiao et al., 2016; Cao et al., 2017). However, currently available FMD vaccines, formulated with a mixture of inactivated viral antigens and oil adjuvants, are intramuscularly administered. Although these oil-adjuvant-based commercial vaccines produce relatively stable immune responses in cattle, they exhibit low antibody titers in pigs compared with that in cattle and high variation in antibody titers between individuals (Lee et al., 2019). Moreover, the inclusion of oil adjuvants can lead to adverse reactions, such as abscesses and fibrosis at the injection site in pigs, resulting in substantial economic losses (Charerntantanakul, 2020). Most commercial vaccines primarily aim to stimulate a humoral immune response, making it challenging to effectively trigger a cellular immune response. If viral infection occurs before antibody titers are reached in the early stages of vaccination, a gap in host defense may occur. Therefore, it is necessary to improve the host’s immune system before the initial antibody formation after vaccination.

Although research on the route of FMDV infection is limited, studies have shown that FMDV initially enters pigs through the oropharynx and may undergo an incubation period in the tonsils or pharynx (Jamal and Belsham, 2013). Ideally, the most effective host defense against respiratory viruses would be to induce a local immune response that neutralizes or eliminates the virus at the site of infection, while simultaneously preventing entry of the virus from the primary site of infection (Mettelman et al., 2022).

The mucosa is present in major exposed areas, such as the oral cavity, respiratory system, digestive system, and urogenital tract, and serves as an important barrier between the internal and external environments in both humans and animals. Mucosa-associated lymphoid tissue (MALT), which regulates immune responses, is present in the mucosa. The MALT is divided into several regions, including gut-associated lymphoid tissue (GALT), bronchus-associated lymphoid tissue (BALT), and nasal-associated lymphoid tissue (NALT), each of which can trigger an immune response to antigens (Brandtzaeg, 2010). This surface is protected from invasion by foreign antigens by an efficient physical barrier consisting of a mucus and glycocalyx layer, and a chemical barrier containing antibacterial agents and secretory immunoglobulin A (sIgA) that responds to specific antigens (Nagashima et al., 2017). The mucosal surfaces possess an advanced immune system, and external stimuli can trigger mucosal immunity that targets specific pathogens. This immune response may also extend to systemic protection, generating antibodies and activating immune cell-mediated mechanisms (Kiyono and Azegami, 2015; Wang et al., 2015).

GALT is the largest lymphoid organ in the host, accounting for approximately 70% of all immune cells (Mörbe et al., 2021). The GALT consists of various lymphoid tissues, including Peyer’s patch (PP) in the small intestine, cecal patch, colonic patch, and isolated lymphoid follicles. These tissues are crucial for mounting an efficient protective immune response (Chen, 2000). Microfold cells (M cells), which are specialized epithelial cells found in the follicle-associated epithelium, are crucial for antigen uptake in GALT and NALT. They play a key role in monitoring immunosurveillance and immunoregulation at mucosal surfaces. M cells express cell surface receptors known as transcytosis, which are responsible for recognizing luminal antigens, actively engulfing these antigens at the apical surface, and exporting them out of the cell through the basolateral plasma membrane (Dillon and Lo, 2019). Additionally, PP constitutively generates germinal centers (GCs) in response to the uptake of microbiome- and food-derived antigens taken up by M cells. The development of GCs is essential for the somatic affinity maturation of B cells that have undergone IgA class switching within PP. Therefore, M cells play a vital role in the regulation and development of sIgA responses (Suzuki et al., 2010; Reboldi and Cyster, 2016; Kobayashi et al., 2019). Immune responses initiated by M cells can induce a diverse array of T helper cells, including Th1, Th2, Th17, and regulatory T cells (Tregs), whereas dendritic cells (DCs) present in PP direct immune responses to specific tissues. To achieve this, T and B cells can imprint homing properties, and B cells can directly control the intestinal microflora using sIgA (Li M. et al., 2020). sIgA is the front-line defense against antigens and pathogens that invade the mucosal surface. It is produced locally, secreted in large quantities, and can neutralize pathogens. Given that pathogens enter the body through the mucosal surface, they play a key role in host defense by inducing protection at the first site of contact between the host and pathogen (Pietrzak et al., 2020).

Glycyrrhizic acid (GA) is a triterpenoid saponin extracted from licorice roots, that has various pharmacological effects. Additionally, GA is non-toxic when ingested and slowly decomposes within the intestines (Wahab et al., 2021). Our previous study showed that a test vaccine with the novel adjuvant GA significantly increased both cellular and humoral immunity compared to those with a commercially available vaccine. Additionally, we confirmed the elevation of several mucosal immunity-related cytokines using qRT-PCR, although the vaccine was intramuscularly administered (Shin et al., 2023).

We aimed to address and overcome the limitations of currently available FMD vaccines by combining intramuscular (I.M.) FMD vaccination with oral administration of the immune-enhancer candidate, GA. We evaluated the efficacy of GA in stimulating mucosal and systemic immunity, and its role as an oral immune enhancer that can be used in combination with a viral vaccine.

## 2 Materials and methods

### 2.1 GA

Glycyrrhizic acid ammonium salt [from glycyrrhiza roots (licorice)] was purchased from Sigma-Aldrich (St. Louis, MO, United States).

### 2.2 Cells

Baby hamster kidney (BHK)-21 (ATCC, Manassas, VA, United States) and fetal porcine kidney (LF-BK, Plum Island Animal Disease Center, Oriten, NY, United States) cells were cultured in Dulbecco’s modified Eagle’s medium (DMEM; HyClone, Logan, UT, United States) supplemented with 5% fetal bovine serum (FBS) and 1% antibiotic-antimycotic (A/A; Gibco, Grand Island, NY, United States) at 37°C in 5% CO_2_. The fetal goat tongue epithelium (ZZ-R 127, Friedrich-Loeffler-Institut, Greifswald-Insel Riems, Germany) cell was cultured in DMEM F12 (HyClone) containing 5% FBS and 1% A/A (Gibco) at 37°C in 5% CO_2_.

### 2.3 Antigen purification and composition of test vaccine

Purified FMDV serotype O (O/PanAsia2, O PA2) and FMDV serotype A (A/SKR/YC/2017, A YC) antigens were prepared by transfecting BHK-21 cells in DMEM containing 1% A/A (Gibco). The virus was inactivated by treating with 0.003 N binary ethyleneimine (BEI) twice for 24 h. The inactivated virus was precipitated using polyethylene glycol 6000 (Sigma-Aldrich). Antigen (146S) was purified through a sucrose density gradient (15–45%), followed by ultracentrifugation at 30,000 rpm for 4 h at 4°C (SW 41Ti, Beckman Coulter, Fullerton, CA, United States) (Lee et al., 2022).

The test vaccine composition administered to mice was as follows: FMDV O PA2+A YC antigen (0.375 + 0.375 μg/dose/100 μL; 1/40 of the pig dose), ISA 206 [(50% w/w); Seppic, Paris, France)], 10% Al(OH)_3_ and 15 μg/dose Quil-A (InvivoGen, San Diego, CA, United States) in a total volume of 100 μL. The test vaccine composition administered to pigs was as follows: FMDV O PA2+A YC antigen (15 + 15 μg/dose/mL), ISA 206 [(50% w/w); Seppic], 10% Al(OH)_3_ and 150 μg/dose Quil-A (InvivoGen) in a total volume of 1 mL.

### 2.4 Combination of oral GA and intramuscular vaccine administration, and safety evaluation through food efficiency and biochemical assays

Mice (6–7 weeks old) were divided into three groups (*n* = 5/group): Negative Control (NC), PBS administration; the Positive Control (PC), an I.M injection of the test vaccine; and experimental (Exp) group, I.M. injection of the test vaccine and oral administration of 100 μg of GA. Pigs (8–9 weeks old) were divided into three groups (*n* = 5–6/group) in the same manner as the experimental mice; the Exp group was orally administered 20 mg/4 mL of GA solution. The GA solution was administered to the mice and pigs via a pipette and zonde (feeding needles), respectively. Following vaccination, GA was administered once daily until 28 days post-vaccination (dpv), and once weekly at the same time each day over a period of 4 weeks. The NC and PC groups were administered equal volumes of PBS instead of GA solution. Mice were intramuscularly injected once into the thigh with 100 μL of the test vaccine (PC and Exp) or PBS (NC). Pigs were intramuscularly injected twice into the neck with 1 mL of the test vaccine (PC and Exp) or PBS (NC) at 28 days intervals. The NC group was administered an equal volume of PBS via the same route. During the experiment, food intake was measured at scheduled times each day and body weight was recorded weekly. Food efficiency ratio (FER) was calculated using the following equation:


$$\text{FER}=\frac{\mathrm{Weight}\,\mathrm{gain}\,\left(\mathrm{grams},g\right)}{\mathrm{Food}\,\mathrm{intake}\,\left(\mathrm{grams},g\right)}\times 100$$

To determine whether the oral administration of GA affects the health (safety) of the target animals, liver and kidney function tests were performed using biochemical assays. The serum levels of alanine aminotransferase (ALT), aspartate aminotransferase (AST) blood urea nitrogen (BUN), creatinine (CREA), lactate dehydrogenase (LDH), total protein (TP), albumin (ALB), and albumin/globulin (A/G) ratio were assessed using HITACHI Automatic Analyzer 3100 (Hitachi High-Tech Corporation, Tokyo, Japan) with test reagents from KLS Bio-Inc. (Gyeonggi-do, Republic of Korea).

### 2.5 Immune response evaluation in mice after combined treatments

To evaluate the efficacy of GA in triggering mucosal immune responses in mice, blood samples were harvested from NC, PC, and Exp mice groups at 0, 7, 14, 21 (early), 28, 35, 42 (mid-term), 56, 70, and 84 (long-term) dpv. Blood samples were collected via retro-orbital bleeding. The serum samples were used to evaluate the antibody titer using structural protein (SP) O, A enzyme-linked immunosorbent assay (ELISA), virus neutralization (VN) titer using a VN test, and sIgA titer using immunoglobulin ELISA. The serum samples were stored at −80°C until further use.

### 2.6 FMDV challenge after co-administration of I.M. FMD vaccination with oral GA administration in mice

To evaluate the protective effect of co-administration of I.M. FMD vaccination with oral GA administration, we performed challenge experiments in mice. Mice were challenged with FMDV (100 LD_50_ of O/VET/2013) at 84 dpv via intraperitoneal (I.P.) injection. Survival rates and changes in body weight of mice were monitored for up to 7 days post-challenge (dpc).

### 2.7 Immune response evaluation in pigs after combined treatments

To evaluate the efficacy of GA in inducing mucosal immune responses in pigs, blood samples were collected at 0, 7, 14, 21 (early), 28, 35, 42 (mid-term), and 56, 70, and 84 (long-term) dpv. The obtained sera were used to evaluate the SP O, A antibody, VN, and sIgA titers. The serum samples were stored at −80°C until further use.

### 2.8 Serological assays (SP ELISA, VN test, secretory IgA ELISA)

To assess the SP antibody levels in the serum samples, ELISA kits specific for FMDV types O and A (PrioCheck™, Prionics AG, Schlieren, Switzerland) were used, following the manufacturer’s instructions. Spectrophotometric readings were obtained at 450 nm by using a spectrophotometer (Hidex, Turku, Finland), and the data were converted to percent inhibition (PI) values. Animals were classified as antibody-seropositive if the PI value of the PrioCheck™ FMDV kit exceeded 50%.

Virus neutralization tests were performed according to WOAH guidelines. Sera from animals were heat-inactivated for 30 min at 56°C. The inactivated sera were then serially diluted 2-fold (from 1:8 to 1:1024). These diluents were exposed to FMDV type O PA2 or A YC at 100 TCID_50_/0.5 mL and incubated for 1 h at 37°C. After incubation, LF-BK cells were treated to all the wells. Cytopathic effects (CPE) were observed after 72 h and titers were calculated and recorded as Log_10_ of the reciprocal of the maximum serum dilution required to neutralize 100 TCID_50_ of the virus.

Serum levels of murine and porcine sIgA were assessed using commercial ELISA kits (CSB-E08413m, CSB-E12063p; Cusabio Inc, Wuhan, China), according to the manufacturer’s protocol. Spectrophotometric readings were obtained at 450 nm by using a spectrophotometer (Hidex) (Lee et al., 2022).

### 2.9 Porcine peripheral blood mononuclear cells (PBMC) isolation, RNA isolation, cDNA synthesis, and qRT-PCR

To elucidate the mechanisms of immune responses triggered by the stimulation of the intestinal mucosa through oral administration of GA, experiments were performed according to previously described protocols (Lee et al., 2022). Porcine PBMCs were extracted from whole blood specimens obtained from pigs (*n* = 5–6/group) used in the experiment at 14 dpv. Briefly, whole blood was collected in heparin-coated tubes (BD, Franklin Lakes, NJ, United States). Porcine peripheral blood mononuclear cells (PBMCs) were isolated by density gradient centrifugation using Lymphoprep (Stem Cell Technologies, Vancouver, BC, Canada). Remaining red blood cells were removed using ammonium–chloride–potassium (ACK) lysing buffer (Gibco). RNA extraction from the purified PBMCs was carried out using TRIzol reagent (Invitrogen, Carlsbad, CA, United States), followed by reverse transcription into cDNA using M-MLV RT (Promega, Madison, WI, United States). The cDNA was amplified on a CFX96 Touch Real-Time PCR system (Bio-Rad, Hercules, CA, USA) using iQ SYBR Green Supermix (Bio-Rad). Gene expression levels were quantified using the delta Ct (ΔCt) method, normalized to the housekeeping gene HPRT, and presented relative to the control values. The primers used in these assays are listed in Supplementary Table 1.

### 2.10 Statistical analysis

Quantitative data were presented as the mean ± SEM, unless otherwise specified. Differences between groups were evaluated using either two- or one-way analysis of variance, followed by Tukey’s or Dunnett’s *post-hoc* analysis. Statistical significance was indicated as *p < 0.05, **p < 0.01, ***p < 0.001, and ****p < 0.0001. Parametric tests were employed to compare the various groups. Survival curves were constructed using the Kaplan–Meier technique, and the long-rank test was applied to evaluate differences between groups. All statistical analyses were performed using Prism 10.0.2 (GraphPad, San Diego, CA, United States).

## 3 Results

### 3.1 Oral GA Administration with I.M. FMD vaccination induces a sustained immune response and potent host defense against FMDV in mice

To confirm the safety of the oral administration of GA, FER was measured using body weight (BW) and food intake changes. No significant changes were observed in BW or FER among the three groups (Supplementary Tables 2, 3).

**FIGURE 1:**
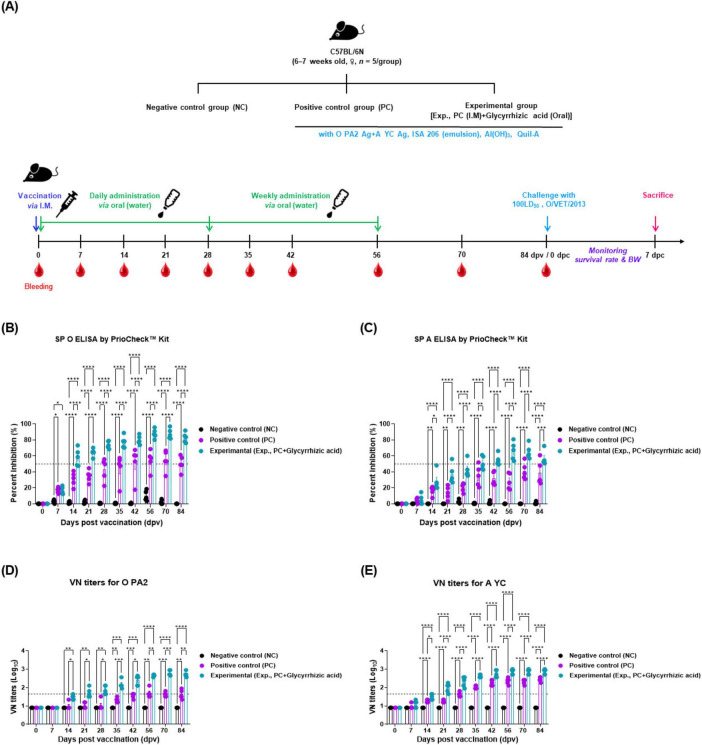
Oral administration of glycyrrhizic acid mediates early, and mid-term, and long-term immune responses in food-and-mouth disease (FMD)-vaccinated mice. Experiments were performed according to the mice experimental strategies described in 2.5, 2.6, 2.9, (see section “2 Materials and methods”) and Figure 1 panel **(A)**. **(A–E)** Experimental strategy **(A)**; SP O antibody titers (PrioCheck™ FMDV kit) **(B)**; SP A antibody titers (PrioCheck™ FMDV kit) **(C)**; VN titers for O PA2 **(D)**; and VN titers for A YC **(E)**. The horizontal dashed lines in panels **(B,C)** represent the manufacturer-specified threshold for a positive result. In panels **(D,E)**, the dashed lines indicate a VN titer of 1:45 (equivalent to 1.65 on a Log_10_ scale), which is considered the minimum level required for protection against viral infection. Data are represented as the mean ± SEM of triplicate measurements (*n* = 5/group). Statistical analyses were performed using two-way ANOVA, followed by Tukey’s *post-hoc* test. *p < 0.05; **p < 0.01; ***p < 0.001; and ****p < 0.0001.

To investigate the effect of oral administration of GA on adaptive immune enhancement in vaccinated mice, the early, mid-term, and long-term immunity of the mice were evaluated (Figure 1A).

The antibody titers measured using SP O and A ELISA were significantly higher in Exp group from 14 to 84 dpv compared with that in the PC group. By 84 dpv, several mice in the PC group failed to maintain positive antibody titers and converted to negative titers. In contrast, all individuals in Exp group maintained positive antibody titers at 84 dpv. The NC group did not show any changes in antibody titers (Figures 1B, C). The animals were considered antibody-positive when the PI value was ≥ 50% for the PrioCheck™ FMDV kit.

The VN titers for O PA2 and A YC, as measured by the VN test, also significantly increased in Exp group, which received oral GA alongside the vaccine from 14 to 84 dpv, with significantly less individual variability than the PC group. The NC group did not show any changes in the VN titers (Figures 1D, E). VN titers with a Log_10_ value greater than or equal to 1.65 are considered capable of protecting the host from FMDV infection.

**FIGURE 2:**
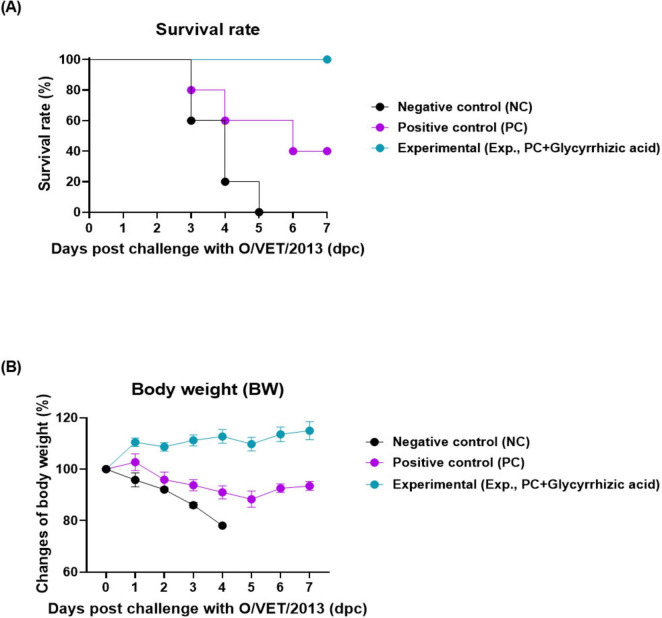
Oral administration of glycyrrhizic acid improves host protection against the virus in food-and-mouth disease (FMD)-vaccinated mice. Experiments were performed according to the mice experimental strategies described in 2.5, 2.7 (see section “2 Materials and methods”) and Figure 1 panel **(A)**. **(A,B)** Survival rates post-challenge with O/VET/2013 **(A)**; Changes in body weight post-challenge with O/VET/2013 **(B)**. Data are presented as mean ± SEM of triplicate measurements (*n* = 5/group).

To investigate the co-administration of GA and FMD vaccine-mediated host protection against viral infections, we conducted a study as depicted in Figure 1A. The Exp group, which was orally administered GA along with vaccination, showed a 100% survival rate. The PC and NC groups showed 40 and 0% survival rates, respectively (Figure 2A). There was no change in body weight in Exp group; however, the PC and NC groups lost more than 10 and 20% of their body weight, respectively (Figure 2B).

### 3.2 Oral GA administration of GA with I.M. FMD vaccination induces systemic immunity and a sustained immune response in pigs

To confirm the safety of oral administration of GA, serum levels of ALT, AST, BUN, CREA, LDH, TP, and ALB and the A/G ratio were measured. Blood biochemical indices were within the normal range, and AST levels in the NC group were significantly higher than those in the Exp group. Except for AST, no significant differences were observed among the Exp, PC, and NC groups (Table 1).

**TABLE 1:** Biochemistry assays in serum of pigs treated with glycyrrhizic acid by oral administration for 84 days post-vaccination.

Group	Days post-vaccina tion (dpv)	ALT (U/L)	AST (U/L)	BUN (mg/dL)	CREA (mg/dL)	LDH (U/L)	TP (mg/dL)	ALB (mg/dL)	A/G ratio
NC	0	41.20 ± 3.68	40.00 ± 2.06	5.90 ± 2.06	0.75 ± 0.02	421.28 ± 45.74	2.58 ± 0.10	2.90 ± 0.06	1.22 ± 0.04
	28	56.80 ± 4.03	56.25 ± 22.48	9.56 ± 1.08	1.01 ± 0.05	496.23 ± 40.97	6.08 ± 0.15	3.36 ± 0.05	1.26 ± 0.08
	56	49.60 ± 1.85	33.40 ± 1.82	10.50 ± 1.21	1.15 ± 0.02	333.10 ± 13.02	6.72 ± 0.15	3.14 ± 0.08	0.89 ± 0.05
	84	47.00 ± 1.74	66.00 ± 14.04^a^	17.26 ± 1.47	1.46 ± 0.05	266.48 ± 2.76	6.06 ± 0.07	3.62 ± 0.04	1.49 ± 0.05
PC	0	37.20 ± 1.73	32.60 ± 0.61	7.50 ± 1.43	0.68 ± 0.02	340.30 ± 13.22	5.34 ± 0.07	2.98 ± 0.07	1.26 ± 0.08
	28	48.40 ± 1.85	45.50 ± 3.32	9.30 ± 0.67	0.93 ± 0.02	456.34 ± 17.62	6.30 ± 0.14	3.32 ± 0.11	1.12 ± 0.07
	56	53.80 ± 3.33	48.60 ± 9.20	13.98 ± 0.95	1.16 ± 0.05	361.90 ± 26.52	6.80 ± 0.10	3.36 ± 0.08	0.98 ± 0.02
	84	43.60 ± 2.79	37.75 ± 25.86	16.78 ± 1.60	1.30 ± 0.07	254.12 ± 8.38	6.34 ± 0.12	3.72 ± 0.09	1.44 ± 0.08
Exp.	0	42.20 ± 1.75	37.40 ± 4,30	7.56 ± 0.91	0.81 ± 0.05	350.48 ± 12.64	5.42 ± 0.08	2.86 ± 0.08	1.12 ± 0.05
	28	55.00 ± 6.94	39.00 ± 4.32	7.30 ± 1.30	1.01 ± 0.04	427.18 ± 16.76	6.58 ± 0.06	3.33 ± 0.18	1.05 ± 0.12
	56	46.00 ± 8.34	33.50 ± 2.08	12.05 ± 2.20	1.22 ± 0.06	402.70 ± 27.63	7.15 ± 0.34	3.25 ± 0.25	0.92 ± 0.16
	84	40.75 ± 3.42	29.50 ± 2.75^b^	13.73 ± 1.41	1.49 ± 0.03	244.05 ± 14.18	6.60 ± 0.20	3.83 ± 0.13	1.40 ± 0.12

The experimental strategy was as described in 2.5 (see section “2 Materials and methods”). Data are represented mean ± SEM of triplicate measurements (*n* = 5–6/group). Statistical analyses were performed using one-way ANOVA, followed by Tukey’s *post-hoc* test. Different superscripts (a,b) represent significant difference at *p* < 0.05. dpv, days post-vaccination; NC, negative control; PC, positive control; Exp, experimental; ALT, alanine aminotransferase; AST, aspartate aminotransferase; BUN, blood urea nitrogen; CREA, creatinine; LDH, lactate dehydrogenase; TP, total protein; ALB, albumin; A/G, albumin-to-globulin ratio.

**FIGURE 3:**
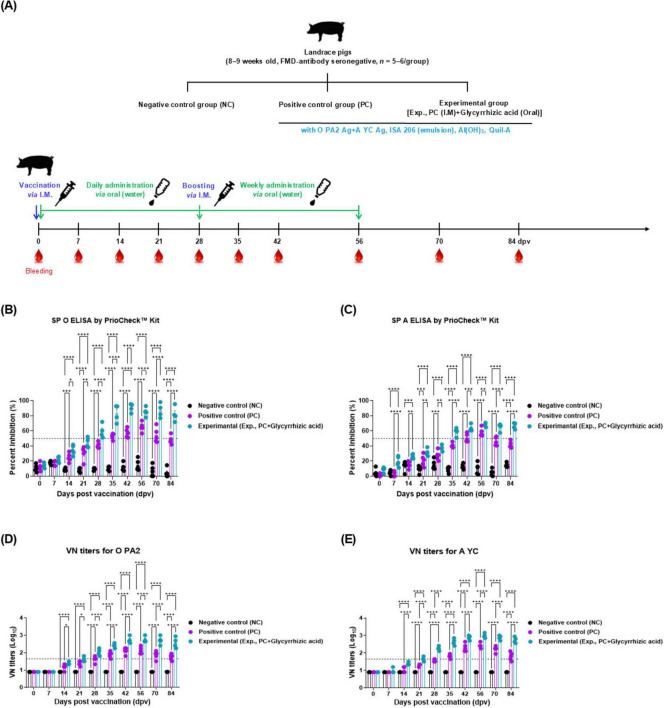
Oral administration of glycyrrhizic acid mediates early, and mid-term, and long-term immune responses in food-and-mouth disease (FMD)-vaccinated pigs. Experiments were performed according to the pigs experimental strategies described in 2.5, 2.8, 2.9, (see section “2 Materials and methods”) and Figure 3 panel **(A)**. **(A–E)** Experimental strategy **(A)**; structural protein (SP) O antibody titers (PrioCheck™ FMDV kit) **(B)**; SP A antibody titers (PrioCheck™ FMDV kit) **(C)**; virus neutralization (VN) titers for O PA2 **(D)**; and VN titers for A YC **(E)**. The horizontal dashed lines in panels **(B,C)** represent the manufacturer-specified threshold for a positive result. In panels **(D,E)**, the horizontal dashed lines denote the VN titer of 1:45 (equivalent to 1.65 on a Log_10_ scale), which is considered the minimum level required for protection against viral infection. Data are represented as the mean ± SEM of triplicate measurements (*n* = 5/group). Statistical analyses were performed using two-way ANOVA, followed by Tukey’s *post-hoc* test. *p < 0.05; **p < 0.01; ***p < 0.001; and ****p < 0.0001.

To assess the effect of the oral administration of GA on vaccination-mediated humoral immunity in pigs, we monitored the early, mid-term, and long-term immune responses in pigs (Figure 3A). When the FMD test vaccine was intramuscularly injected and GA orally administered to the pigs, the antibody titers measured using SP O and A ELISA significantly increased in Exp group from 7 (SP A ELSA) or 14 (SP O ELISA) dpv to 84 dpv, compared with those in the PC group. Antibody titers in the NC group did not show any differences (Figures 3B, C). The animals were considered antibody-positive when the PI value was ≥ 50% for the PrioCheck™ FMDV kit.

The VN titers for O PA2 and A YC also significantly increased from 14 to 84 dpv in Exp group after the oral administration of GA compared with that in the PC group. From 56 dpv, the VN titers of the PC group decreased to 84 dpv, whereas those of Exp group maintained a high VN titer. VN titers in the NC group did not change (Figures 3D, E). VN titers with a Log_10_ value greater than or equal to 1.65 are considered capable of protecting the host from FMDV infection.

### 3.3 Oral GA administration with I.M. FMD vaccination increases sIgA expression in the serum of mice and pigs

**FIGURE 4:**
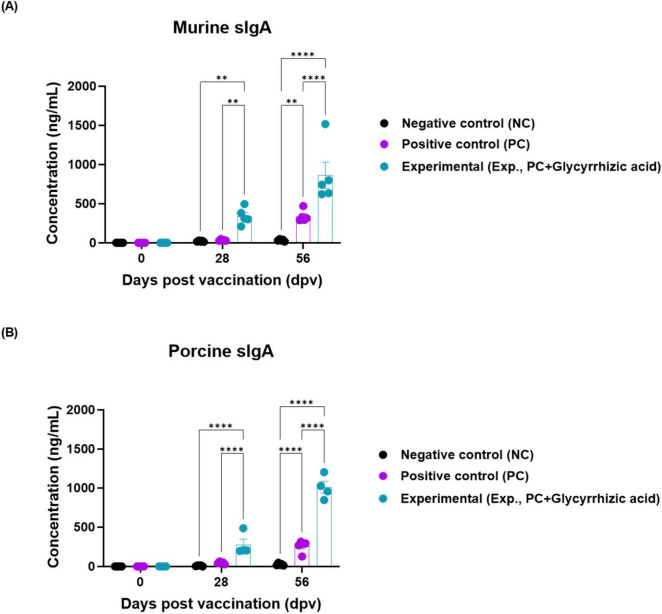
Oral administration of glycyrrhizic acid increases the expression of secretory IgA. Experiments were performed according to the food-and-mouth disease (FMD)-vaccinated mice and pigs experimental strategies described in 2.5, 2.6, 2.8, 2.9, (see section “2 Materials and methods”), Figures 1, 3 panel **(A)**. The concentration of murine and porcine secretory IgA (sIgA) were assessed in serum using a secretory IgA ELISA kit. **(A,B)** sIgA antibody titers in mice (Cusabio secretory IgA ELISA kit) **(A)**; sIgA antibody titers in pigs (Cusabio secretory IgA ELISA kit) **(B)**. Statistical analyses were performed using two-way ANOVA, followed by Tukey’s *post-hoc* test. **p < 0.01; and ****p < 0.0001.

To evaluate the mucosal immunity induced by the oral administration of GA, we measured sIgA levels in serum derived from mice and pigs. The level of sIgA significantly increased at 28 and 56 dpv in Exp group compared with that in the PC and NC groups in mice and pigs (Figures 4A, B).

### 3.4 Oral GA administration with I.M. FMD vaccination stimulate systemic immune responses via cytokine gene expression in pigs

**FIGURE 5:**
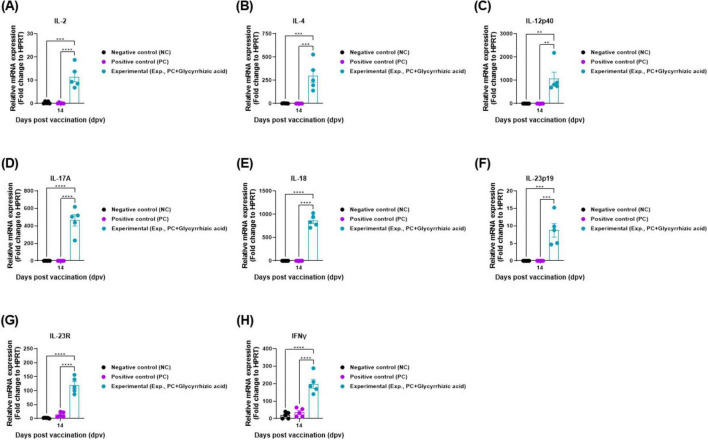
Oral administration of glycyrrhizic acid induces the gene expression of systemic immune-related cytokines in food-and-mouth disease (FMD)-vaccinated pigs. Experiments were performed according to the pigs experimental strategies described in 2.5, 2.8, 2.10, (see section “2 Materials and methods”) and Figure 3 panel **(A)**. **(A–H)** Gene expression levels of *IL-2* **(A)**; *IL-4* **(B)**, *IL-12p40* **(C)**; *IL-17A* **(D)**; *IL-18* **(E)**; *IL-23p19* **(F)**; *IL-23R* **(G)**; and *IFNγ* **(H)**. Statistical analyses were performed using two-way ANOVA, followed by Tukey’s *post-hoc* test. *p < 0.05; **p < 0.01; ***p < 0.001; and ****p < 0.0001.

To elucidate the GA-mediated systemic immunity, cytokine gene expression was measured by qRT-PCR in porcine PBMCs, as shown in Figure 3A. The level of cytokines, such as IL-2, IL-4, IL-12p40, IL-17A, IL-18, IL-23p19, IL-23R, and IFNγ in the serum of the Exp group was significantly higher than that in the PC group at 14 dpv (Figures 5A–H).

## 4 Discussion

The infection and spread of FMD are influenced by the interactions between the pathogen, host, and environment (Jones et al., 2008). Vaccination is widely known to be an effective preventive tool against pathogenic infections. However, to prevent infection and transmission, the health status and the innate immune system of the host must also be considered. The weakened immune status of the host and rapid changes in the environment can create new transmission patterns, allowing pathogens to invade the host, making it more susceptible to infection. To overcome this, it is essential to improve both individual and collective health, thereby preventing the spread of pathogens and controlling group infections (Engering et al., 2013).

GA, the most important active component of licorice, is known for its immunomodulatory, hepatoprotective, and pharmacological effects (Celik et al., 2012; Pastorino et al., 2018). Recent studies have indicated its potential as a drug stabilizer and anticancer agent. Owing to its hydrophilic and lipophilic properties, GA can interact with cell membranes, facilitating the intracellular entry of other drugs, making it a potential drug delivery system (Hogstrand, 2015; Zhang et al., 2023). Moreover, GA has been deemed safe for human and animal consumption at established safe dosage levels, and is a flavoring substance, allowing its unrestricted use in food products and making it suitable for research purposes (Van Gelderen et al., 2000; Selyutina and Polyakov, 2019). Therefore, we investigated whether oral GA administration in conjunction with I.M. FMD vaccination could stimulate the mucosal and systemic immune systems in the host, thereby enhancing and prolonging the efficacy of the vaccine.

We evaluated adaptive immunity to determine whether the oral administration of GA could enhance the effectiveness of an inactivated FMD vaccine by inducing mucosal immune responses in mice. There was no significant difference in the FER of mice in the Exp, PC, and NC groups, as evaluated by measuring body weight and food intake. Therefore, oral administration of GA did not have a negative effect on food intake and growth (weight gain) of the experimental animals (Supplementary Tables 1, 2). The combination of oral GA administration and I.M. FMD vaccination significantly increased SP antibody and VN titers in Exp group with less individual variation (Figure 1). Co-administration of oral GA and I.M. FMD vaccine elicited host protection against FMDV infection in mice (Figure 2). This study demonstrated that the oral administration of GA stimulated the mucosal immune system, enhanced individual immunity, and improved the efficacy of the FMD vaccine.

Subsequently, we performed follow-up experiments to evaluate the effects of oral GA administration on the adaptive immune response in pigs and determine whether it activated intestinal mucosal immunity. Biochemical analyses were performed on serum samples collected at 0, 28, 56, and 84 dpv to evaluate the safety of GA in pigs. No significant differences were found in the levels of all parameters except for AST (Table 1). AST is released into the blood when liver or muscle damage occurs and its blood concentration increases (Lala et al., 2023). These results confirmed that the oral GA administration dose used in our experiments did not have any negative effects on pigs.

The antibody and VN titers were higher in Exp group than in the PC group from the early stages, with significant differences observed from 35 to 84 dpv (Figure 3). Levels of sIgA, a marker of mucosal immunity, were also higher in the Exp group than that in the PC group (Figure 4). These results revealed that oral GA administration effectively induces adaptive immune responses through simultaneous induction of mucosal and systemic immune responses.

To define the basic mechanism of systemic immune response induced by oral GA administration, *IL2, IL-4, IL-12p40, IL-18, IL-17A, IL-23p19, IL-23R*, and *IFNγ* gene expression levels were quantified. Significantly higher gene expression was detected in Exp group than in the PC group, indicating that oral administration of GA can trigger a potent systemic immune response (Figure 5).

GALT, which is responsible for major immune responses in the gut, includes PP. PP are located in the subepithelial dome region and contain myeloid DCs that induce Th2 cell differentiation, lymphoid DCs that induce Th1 cell differentiation, and double-negative DCs. Myeloid DCs also mediate the differentiation of Th3 and Tregs after exposure to food antigens (Banchereau et al., 2000; Iwasaki and Kelsall, 2000, 2001). Moreover, M cells in PP selectively take up antigens from the external environment, transport them across the epithelial barrier, and deliver them directly to subepithelial DCs, which then present antigens locally to adjacent mucosal T-cell areas (Neutra and Kozlowski, 2006). Mucosal T cells that receive antigen presentation express IL-12, a key cytokine that initiates Th1 cell differentiation, and IL-4, which induces Th2 differentiation through STAT6 (Ruterbusch et al., 2020; Ma et al., 2021). Subsequently, differentiated Th1 cells express IFNγ and IL-2, whereas Th2 cells express IL-4 and IL-10 (Zhou et al., 2021). Previous studies have shown that IL-4 and IL-10 synergistically induce substantial changes in the IgA isotype production. Additionally, these cytokines promote the class switching of B cells to IgA and their differentiation into sIgA-producing cells (Asano et al., 2004; Li Y. et al., 2020). Therefore, IL-4 and IL-10 can be considered as critical cytokines in the generation of sIgA.

Notably, oral GA administration can activate mucosal-associated invariant T (MAIT) cells which are crucial in the initial defense against pathogenic bacteria and yeast on mucosal surfaces and play an important role in maintaining mucosal barrier homeostasis (Nel et al., 2021). Previous studies have reported that MAIT cells are found in the oral mucosa and respiratory tract and can be activated by antigen-presenting cells (APCs), such as DCs, macrophages, and monocytes (Reantragoon et al., 2013; Chen et al., 2017; Sobkowiak et al., 2019). MAIT cell activation is restricted by MR1 on APCs and triggered by microbial non-peptide ligands (Meierovics et al., 2013). Upon antigen invasion, activated APCs produce cytokines, such as IL-12 and IL-18, and the activated MAIT cells, can produce pro-inflammatory cytokines, such as IFNγ, TNFα, while MAIT cells, once activated, release pro-inflammatory cytokines such as IL-18R, IL-12R, and IL-23R, and MR1, thereby supporting local immune responses and directly stimulating innate immunity (Ussher et al., 2014). Notably, IL-17A, in conjunction with IL-23, is an important cytokine in the formation of neutrophil extracellular traps (NETs). NETs consist of extracellular DNA associated with antimicrobial proteins derived from neutrophil granules and nuclei, and capture various microbes, providing a crucial innate immune mechanism (Brinkmann et al., 2004). Therefore, NET plays a crucial role in preventing viral invasion into the host at mucosal sites.

The novel vaccination program combining oral GA administration and I.M. FMD vaccination proposed in this study demonstrated that multifaceted immune response enhancement can improve protection against FMDV. Specifically, the program increased SP antibodies and VN titers in both experimental and target animals, and enhanced the ability to rapidly capture viruses penetrating the mucosal barrier via NETs, thereby reducing the risk of host infection. Additional benefits of GA, such as economic feasibility, accessibility, and effectiveness in improving pork quality, further enhance the practicality of this program (Bazekin et al., 2023). In particular, the fact that GA can be used as a feed additive or a component of bait vaccines may increase the efficiency of disease prevention and control.

Building on the previous study that demonstrated the potential of GA as an adjuvant for I.M. vaccination, this study confirmed the efficacy of GA as an oral adjuvant (Shin et al., 2023). However, there are some limitations to this study, including the inability to observe the immune response at mucosal sites and the primary focus on humoral immunity within the adaptive immune response. Furthermore, while serum sIgA and mucosal sIgA differ significantly in their functions and roles, the increase in mucosal immune-related mRNA levels and the observed increase in serum sIgA expression are notable. Activation of the mucosal immune system may impact not only local immune responses but also systemic immunity. The increase in serum sIgA could be an indirect result of mucosal immune system activation, suggesting that plasma cells generated in the GALT may have migrated into the systemic circulation (Keppler et al., 2021).

Therefore, our hypothesis that oral administration of GA enhanced the efficacy of FMD vaccine is based on these evidences of complex immune responses. This suggests that mucosal immune stimulation may lead to enhancement of not only local defense but also systemic immune responses, which may have enhanced the overall defense capability against FMDV.

However, this interpretation requires further experimental validation. Future studies are planned to address these limitations. Specifically, we will directly observe the immune response at mucosal sites, with a particular focus on sIgA expression. Additionally, we plan to more precisely assess the efficacy of oral GA administration by using a vaccine with a reduced antigen dose or fewer vaccinations. We also aim to evaluate the preventive effects of GA as a livestock feed additive against various veterinary diseases. Through this comprehensive approach, this study demonstrates that a novel vaccination program utilizing GA could serve as an effective and practical strategy for protection against FMD, confirming its potential as an oral adjuvant for future oral FMD vaccines.

## Data Availability

The original contributions presented in this study are included in this article/Supplementary material, further inquiries can be directed to the corresponding author.
