# Supplementary material for: Oral administration of glycyrrhizic acid with intramuscular injection of foot-and-mouth disease vaccine enhances the adaptive immune system
Source: Front Microbiol. 2025 Feb 19;16:1502630. doi: 10.3389/fmicb.2025.1502630 (PMC11880001; doi:10.3389/fmicb.2025.1502630)
Supplement: Supplementary file 1 [file Data_Sheet_1.pdf]

Supplementary Material

Oral Administration of Glycyrrhizic Acid with Intramuscular Injection of Viral Vaccine Enhances the Adaptive Immune System

Seokwon Shin, Hyeong Won Kim, Mi-kyeong Ko, So Hui Park, Jong-Hyeon Park, Su-Mi Kim, Min Ja Lee*

Center for Foot-and-Mouth Disease Vaccine Research, Animal and Plant Quarantine Agency, Gimcheon-si 39660, Gyeongsangbuk-do, Republic of Korea

*** Correspondence:**
Min Ja Lee
herb12@korea.kr

1 Supplementary Tables

1.1 Supplementary Table

Supplementary Table 1. List of primer sequences for qRT-PCR.

Target	Forward/Reverse	Sequence (5′-3′)	Length (mer)
IL-2	IL-2 F	AAGCTCTGGAGGGAGTGCTA	20
	IL-2 R	CAACAGCAGTTACTGTCTCATCA	23
IL-4	IL-4 F	CTCACCTCCCAACTGATCCC	20
	IL-4 R	TGTGTCCGTGGACGAAGTTG	20
IL-12p40	IL-12p40 F	GGAGTATAAGAAGTACAGAGTGG	23
	IL-12p40 R	GATGTCCCTGATGAAGAAGC	20
IL-17A	IL-17A F	CTCGTGAAGGCGGGAATCAT	20
	IL-17A R	GGTGTGCTCCGGTTCAAGAT	20
IL-18	IL-18 F	AGCTGAAAACGATGAAGACCTG	22
	IL-18 R	AAACACGGCTTGATGTCCCT	20
IL-23p19	IL-23p19 F	CCATATCCAGTGCGGGGATG	20
	IL-23p19 R	AGGCCTTGGTGGATCCTTTG	20
IL-23R	IL-23R F	TCCCTCATTGCAAAGCACAA	20
	IL-23R R	GCATCTCCTCTTGCAAGCAAAT	22
IFNγ	IFN-γ F	GCCATTCAAAGGAGCATGGAT	21
	IFN-γ R	CTGATGGCTTTGCGCTGGAT	20
HPRT	HPRT F	CCCAGCGTCGTGATTAGTGA	20
	HPRT R	GCCGTTCAGTCCTGTCCATA	20

Supplementary Table 2. Body weight gain of mice treated with or without glycyrrhizic acid by oral administration for 56 days post vaccination (dpv).

Group	0 dpv	7 dpv	14 dpv	21 dpv	28 dpv	56 dpv
**NC**	18.68±0.28	19.93±0.38	20.85±0.42	21.39±0.43	21.54±0.31	23.13±0.60
**PC**	19.10±0.34	20.84±0.31	21.00±0.49	21.57±0.45	21.70±0.46	24.04±0.36
**Exp.**	19.33±0.14	20.93±0.15	21.99±0.38	22.60±0.34	22.31±0.43	23.61±0.33

Experiments were performed according to the mice experimental strategies described in **2.5**, **2.6**, **2.9**, (**Materials and Methods** section) and **Figure 1A**. Data are represented as the mean ± SEM of triplicate measurements (n = 5/group). Statistical analyses were performed using two-way ANOVA, followed by Tukey's *post-hoc* test.

dpv, days post-vaccination; NC, negative control; PC, positive control; Exp., experimental

Supplementary Table 3. Weight gain, food intake, and food efficiency ratio (FER) of mice treated with glycyrrhizic acid by oral administration for 56 days post vaccination.

Group	Weight gain (g/56 dpv)	Food intake (g/56 dpv)	FER
**NC**	4.45±0.61	121.57±3.45	3.66±0.50
**PC**	4.85±0.58	152.86±2.68	3.17±0.38
**Exp.**	4.29±0.37	126.71±2.73	3.38±0.29

Experiments were performed according to the mice experimental strategies described in **2.5**, **2.6**, **2.9**, (**Materials and Methods** section) and **Figure 1A**. FER, food efficiency ratio {FER=Body weight gain (g/dpv) / food intake (g/dpv) *100}. Data are represented as the mean ± SEM of triplicate measurements (*n* = 5/group). Statistical analyses were performed using two-way ANOVA, followed by Tukey's *post-hoc* test.

dpv, days-post vaccination; FER, food efficiency ratio; NC, negative control; PC, positive control; Exp., experimental
